# Epidemiologic Evaluation of Measurement Data in the Presence of Detection Limits

**DOI:** 10.1289/ehp.7199

**Published:** 2004-09-13

**Authors:** Jay H. Lubin, Joanne S. Colt, David Camann, Scott Davis, James R. Cerhan, Richard K. Severson, Leslie Bernstein, Patricia Hartge

**Affiliations:** ^1^Division of Cancer Epidemiology and Genetics, National Cancer Institute, Bethesda, Maryland, USA; ^2^Southwest Research Institute, San Antonio, Texas, USA; ^3^Fred Hutchinson Cancer Research Center and the University of Washington, Seattle, Washington, USA; ^4^Mayo Clinic, College of Medicine, Rochester, Minnesota, USA; ^5^Karmanos Cancer Institute and Department of Family Medicine, Wayne State University, Detroit, Michigan, USA; ^6^Department of Preventive Medicine, Norris Comprehensive Cancer Center, Keck School of Medicine, University of Southern California, Los Angeles, California, USA

**Keywords:** dust, environmental exposure, imputation, missing data, non-Hodgkin lymphoma, pesticides

## Abstract

Quantitative measurements of environmental factors greatly improve the quality of epidemiologic studies but can pose challenges because of the presence of upper or lower detection limits or interfering compounds, which do not allow for precise measured values. We consider the regression of an environmental measurement (dependent variable) on several covariates (independent variables). Various strategies are commonly employed to impute values for interval-measured data, including assignment of one-half the detection limit to nondetected values or of “fill-in” values randomly selected from an appropriate distribution. On the basis of a limited simulation study, we found that the former approach can be biased unless the percentage of measurements below detection limits is small (5–10%). The fill-in approach generally produces unbiased parameter estimates but may produce biased variance estimates and thereby distort inference when 30% or more of the data are below detection limits. Truncated data methods (e.g., Tobit regression) and multiple imputation offer two unbiased approaches for analyzing measurement data with detection limits. If interest resides solely on regression parameters, then Tobit regression can be used. If individualized values for measurements below detection limits are needed for additional analysis, such as relative risk regression or graphical display, then multiple imputation produces unbiased estimates and nominal confidence intervals unless the proportion of missing data is extreme. We illustrate various approaches using measurements of pesticide residues in carpet dust in control subjects from a case–control study of non-Hodgkin lymphoma.

Epidemiologic studies often collect quantitative measurement data to improve precision and reduce bias in exposure assessment and in the estimation of the effect of exposure on risk of disease, as measured by odds ratios (Hatch and Thomas 1993; Sim 2002). Some measurements serve as biomarkers for “dose”—for example, residual radiation in tooth enamel as a marker of exposure to ionizing radiation (Desrosiers and Schauer 2001)—whereas other measures are more indirect—for example, urinary cotinine level as an indicator of exposure to environmental tobacco smoke (Woodward and Al Delaimy 1999). Problems in the analysis of measurement data commonly arise because measurement procedures often have detection limits (DLs). A DL may represent a floor value, a ceiling value, or an interval where precise quantitative levels cannot be determined. For example, exposure assessment for nuclear workers relied on radiation film badges that record radiation levels only above a fixed minimum, because of limits in film photosensitivity (Gilbert et al. 1996; Kerr 1994). Investigators encountered ceiling levels of particle-bound polycyclic aromatic hydrocarbons in rural Chinese dwellings when values exceeded 60,000 ng/m^3^, the upper limit of the measurement protocol (Ligman et al. 2004).

Although values below or above a DL are “missing,” data are not missing at random in the usual sense, because their absence reflects levels of exposure. This type of missing data is called “nonignorable missing,” and the simple exclusion of such “interval-measured” data can bias results (Little and Rubin 1987; Schafer 1997).

Analytic procedures for environmental measurement data with DLs are often presented in the context of environmental monitoring where the primary goal is estimation of distributional parameters when numbers of measurements are limited (Gleit 1985; Haas and Scheff 1990; Helsel 1990; Persson and Rootzen 197; Singh and Nocerino 2002; Travis and Land 1990). In epidemiologic studies, measurement data are used to characterize exposures of study subjects and are typically employed in two ways: *a*) to develop regression models to examine the relationship between a measured value (dependent variable) and covariates (independent variables); and *b*) as covariates in a risk analysis to estimate the relationship between a binary disease outcome and exposure measures and other factors. In this article, we focus on the first application, namely, the regression of an exposure measurement on covariate factors. The use of measurements with DLs in risk regression will be considered in another article.

Investigators apply various strategies for measurement data with DLs, including replacement of measurements below a DL with a single value, for example, DL, DL/2, or DL/√2 (Helsel 1990; Hornung and Reed 1990). Less frequently, measurements below a DL are assigned a value of zero. Unless such measurements indicate a true zero exposure, this latter strategy clearly distorts results and is not considered further in this article. If the distribution of the measurement data is known—for example, measurements are log-normally distributed—then an alternative strategy replaces values below the DL with expected values of the missing measurements, conditional on being less than the DL (Garland et al. 1993; Gleit 1985). For measurement *Z* and detection limit DL, we denote this value *E*[*Z* |*Z* < DL]. Calculation of the conditional expected value requires the investigator to either know or estimate parameters of the measurement distribution.

Substitution schemes like those described above are simple, because one value replaces all measurements below the DL, and, except for *E*[*Z*|*Z* < DL], distributional assumptions are not considered. However, because a single value represents all measurements below the DL, parameter estimates and their variances are likely biased, unless the proportion is small, which potentially distorts inference. This limitation led to a single-impute “fill-in” method (Helsel 1990; Moschandreas et al. 2001a, 2001b). An investigator first characterizes the form of the distribution and estimates its parameters and then assigns randomly sampled values below the DL from the estimated distribution. Fill-in values along with measured values above the DL are then used in analyses. With appropriate estimation techniques, this approach accommodates multiple DLs.

As described by Helsel (1990) and applied by Moschandreas et al. (2001b), the fill-in method did not include complex modeling of regression factors. In addition, although the fill-in approach assigned random values from an appropriate distribution, it did not account for the variability of the imputation process, because the inserted values are not real data. In this article, we illustrate methods for epidemiologic data that account for measurements with DLs, using data from a case–control study of non-Hodgkin lymphoma (NHL) (Colt et al. 2004). The example evaluates the relationship between concentrations of pesticide analytes in carpet dust and use of pesticide products in and around the home. We restrict analysis to control subjects, with adjustment for study design factors.

## Example Data from a Case–Control Study of NHL and Pesticides

The principal exposure of the general population to pesticides occurs in the home (Nigg et al. 1990) as the result of indoor use, track-in or drift from outdoors, intrusion of vapors from foundation treatments, or take-home contamination from occupational use (Bradman et al. 1997; Lewis et al. 1999, 2001). Pesticide residues are retained in carpets, migrating into the underlying foam pad, and may persist for months or years.

### Data source.

We consider data from controls from a multicenter, population-based case–control study of NHL, conducted in the United States: the Detroit, Michigan, metropolitan area; the state of Iowa; Los Angeles County, California; and the Seattle, Washington, metropolitan area (Colt et al. 2004). Controls include 1,057 residents 20–74 years of age, frequency matched to cases on age, sex, race, and study area, with an oversampling of African-American subjects in Los Angeles and Detroit.

Interviewers collected information from respondents on lifetime residential history and the frequency and form of pesticides used to treat various types of pests (e.g., flying insects, crawling insects, lawn weeds). Interviewers obtained vacuum cleaner bags from 95% of subjects who had used their vacuum cleaners within the past year and had owned at least half of their carpets or rugs for 5 years or more. Bags were shipped in insulated boxes by overnight mail to Southwest Research Institute and placed in freezers. Samples were collected and analyzed for 513 control subjects.

### Measurement of carpet dust.

The protocol for the collection and measurement of dust samples has been described previously (Colt et al. 2004). Briefly, before extraction and analysis, dust samples were sieved through a 100-mesh sieve to obtain the fine (< 150 μm) dust. Neutral extractions were carried out for 25 pesticides (18 insecticides, six herbicides, and *ortho*-phenylphenol), seven polycyclic aromatic hydrocarbons, and five polychlorinated biphenyl congeners. Acid extractions were carried out for four herbicides and pentachlorophenol. Extracts were analyzed using gas chromatography/mass spectrometry (GC/MS) in selected ion monitoring mode. Analyte amounts were quantified using the internal standard method. In the full study, GC/MS analysts were blinded to disease status.

After analyzing about half of the samples, investigators began monitoring additional ions for some neutral analytes to clarify identification at low levels, resulting in raised DLs for 14 pesticides. DLs were also raised when < 2 g dust were available. An additional problem with some dust samples involved the presence of interfering compounds (i.e., compounds that coeluted with the target analyte), creating uncertainty and prohibiting assignment of specific concentrations.

For three scenarios analysts could provide concentrations only within an interval, which we accommodated by defining a lower bound (LB) and an upper bound (UB) of possible values. If the analyte was not detected and no interferences were present (type I), the LB was set to zero and the UB was set to the specified DL. If there was an interfering compound but insufficient evidence for the presence of the target analyte (type II), the GC/MS analyst reported the result as a nondetect with a DL equal to the entire peak of the coeluting compounds. We set the LB to zero and the UB to 20% of the raised peak or to the DL, whichever was larger. If the target analyte and the interference were both present (type III), the analyst reported an “elevated detect” with a concentration equal to the entire peak of the coeluting compounds. We set the LB bound to the maximum of 20% of the recorded peak, or the DL, and the UB to the maximum of 90% of the reported peak, or the DL, resulting in an interval bounded away from zero.

For ease of presentation, we allow the replacement of measurements below the DL with DL/2 (which applies to missing data types I and II) to refer more generally to the replacement with (LB + UB)/2 (which applies to missing data types I, II, and III).

## Methods and Analysis

Preliminary analysis indicates that measurement data are consistent with a log-normal distribution. If *Z* denotes the measured value of an analyte and is log-normally distributed, denoted *Z* ~ LN(μ,σ^2^), then by definition log(*Z*) is a normal random variable with mean μ and variance σ^2^, denoted log(*Z*) ~ *N*(μ,σ^2^) (Singh et al. 1997). Suppose *X* = (*X*_0_, … , *X_K_*)*^t^* is a column vector of covariates, with *X*_0_ = 1, and β= (β_0_, … , β*_K_*)*^t^* is a column vector of regression parameters, where *t* denotes vector transpose. If data are complete, then a linear regression equation has the form log(*Z*) = β*^t^X* + ɛ, where ɛ~ *N*(0, σ^2^). For each *X*, the model implies that *Z* is log-normally distributed with mean β*^t^X*; that is, *Z* ~ LN(β*^t^X*, σ^2^).

### Regression analysis in control data.

We evaluate the association between analyte concentration and pesticide use by fitting a linear regression model of the logarithm of the analyte level on subject characteristics. Regression (independent) covariates include indicator variables for season of sample collection, presence of oriental rugs, study center, sex, age (< 45, 45–64, ≥ 65 years), race (African American, Caucasian, other), type of home (single family, townhouse/duplex/apartment, other), year of home construction (< 1940< 1940–1959–1960–1979, ≥ 1980), and educational level (< 12, 12–15, ≥ 16 years). As in Colt et al. (2004), covariates vary slightly with analyte. Models also include five variables describing the use of insect treatment products: ever/never used products to treat for crawling insects, flying insects, fleas/ticks, termites, and lawn/garden insects. We use data from current homes only.

Regression analysis is hampered by the presence of measurements known only within bounds. We assume that the probability distributions of measurements below the DL (more precisely, within the LB and UB interval) depend only on observed data; that is, the interval-measured concentrations arise from the same distributions that generate the measured values. Let *F*(•) be the cumulative distribution function and *f*(•) the probability density function for a log-normal random variable. Suppose *X_i_* = (*X_i_*_0_, … ,*X_iK_*)*^t^* is the covariate vector for the *i*th of *i* = 1, … , *n* subjects. LB*_i_* and UB*_i_* are recorded for *i* = 1, … , *n*_0_ individuals, whereas a specific *Z_i_* measurement is recorded for *i* = *n*_0_ + 1, … , *n*_0_ + *n*_1_ individuals. LB and UB are subscripted to allow different DLs. Using a Tobit regression approach (Gilbert 1987; Persson and Rootzen 1977; Tobin 1958), the log-likelihood function has the form


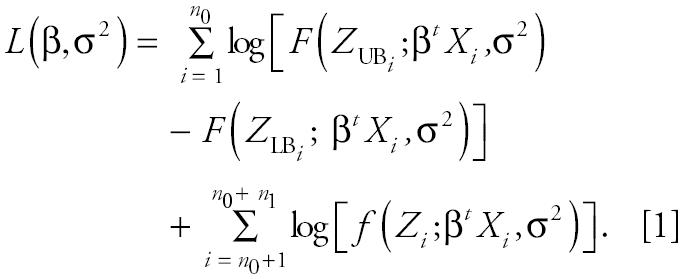


The first summand derives from the *n*_0_ interval measured values and involves the difference of the cumulative distribution function *F* evaluated at UB and at LB; that is, the probability the measurement lies between the LB and UB. The second summand derives from the *n*_1_ detected values. Maximum likelihood estimates (MLEs) for β and their covariance matrix are obtained by maximizing Equation 1 and computing the inverse information matrix using standard methods.

### Imputation of missing concentrations.

If the goal is to evaluate pesticide use and analyte levels in carpet dust, represented by the β parameters, then the Tobit regression of Equation 1 is sufficient and no imputation is required. For further analysis or for graphical display, it is useful to generate values for measurements below DLs. We consider several different approaches, including inserting DL/2, inserting *E*[*Z*|*Z* < DL], or using a single or multiple imputation (Little and Rubin 1987).

A multiple imputation procedure is carried out as follows. Using all data (measured concentrations, missing data types I–III, and covariates), we create the log-likelihood function 1, solve for the MLEs of β and σ^2^ (denoted β̂ and ς̂^2^), and impute a value by randomly sampling from a log-normal distribution with the estimated parameters. However, in selecting fill-in values we cannot ignore that β̂ and ς̂^2^ are themselves estimates with uncertainties. We therefore do not use β̂ and ς̂^2^ for the imputation, but rather β̃ and σ̃^2^, which are estimated from a bootstrap sample of the data (Efron 1979). Bootstrap data are generated as described below by sampling with replacement, and represent a sample from the same universe as the original data. We repeat the process to create multiple data sets, which are then independently analyzed and combined in a way that accounts for the imputation. Differences in regression results in the multiple data sets reflect variability due to the imputation process.

This procedure, however, omits a source of variability. We have tacitly assumed that the LB and UB are fixed and known in advance. When there are no interfering compounds (missing type I), the assumption is justified because the DL is determined before the GC/MS dust analysis. When there are interfering compounds (missing types II and III), the assumption cannot be fully justified because the bounds depend on the amount of interference and therefore are random. In the NHL data, we assume this uncertainty is small relative to other uncertainties. The imputation proceeds as follows:

Step 1: Create a bootstrap sample and obtain estimates β̃ and σ̃^2^ based on Equation 2. Bootstrap data are generated by sampling with replacement *n* times from the *n* subjects. Sampling “with replacement” selects one record at random and then “puts it back” and selects a second record. After *n* repetitions, some subjects are selected multiple times, whereas other subjects are not selected at all. If *w_i_* is the number of times the *i*th subject is sampled, then the log-likelihood function for the bootstrap data is


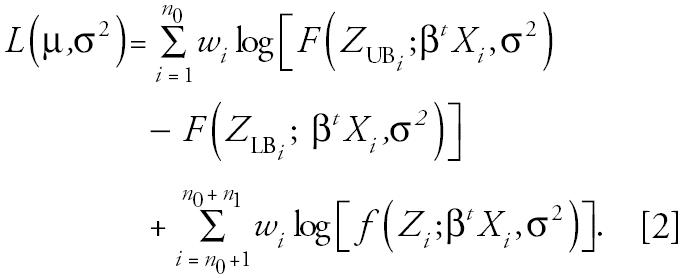


Step 2: Impute analyte values based on sampling from LN (β̃*^t^*X, σ̃^2^). For the *i*th subject, assign the value


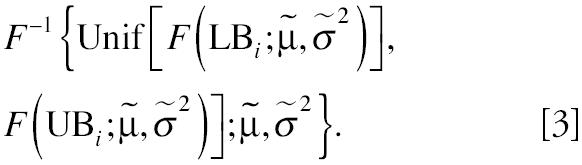


This quantity consists of various elements. *F*(*LB_i_*; β̃*^t^X*, σ̃^2^) and *F*(UB_i_; β̃*^t^X*, σ̃^2^) are the cumulative probabilities at UL*_i_* and UB*_i_*, respectively, based on parameters β̃, σ̃^2^. Both values lie between zero and one. Select randomly from a uniform distribution on the interval [*a*, *b*], denoted Unif[*a*, *b*], in particular the interval [*F*(*LB_i_*; β̃*^t^X_i_*, σ̃^2^), *F*(UB_i_; β̃*^t^X_i_*, σ̃^2^)]. The inverse cumulative distribution function, *F*^−1^(•), is the required imputed value in original units between LB*_i_* and UB*_i_*. Repeat using the same β̃, σ̃^2^ for each missing value. Detected values are not altered.

Step 3: Repeat steps 1 and 2 to create *M* plausible (or “fill-in”) data sets. Remarkably, *M* need not be large, and a recommended value is between 3 and 5, with larger values if greater proportions of data are missing (Little and Rubin 1987; Rubin 1987). We select *M* = 10 to fully account for the variance from the imputation.

Step 4: Fit a regression model to each of the *M* data sets and obtain *M* sets of parameter estimates and covariance matrices. Combine the *M* sets of estimates to account for the imputation (Little and Rubin 1987; Schafer 1997). The imputation procedure results in confidence intervals (CIs) that are wider than the single-imputation, fill-in approach.

### Simulation study.

We conducted a simulation study, using a simple regression model with zero intercept and no covariates, to evaluate the imputation approaches, the effects of the proportion of data below the DL, and sample size. We generated data sets of size *n* by sampling from a log-normal distribution with parameters (μ,σ^2^), and defined the DL such that in expectation *p* percent of the samples falls below the DL; that is, DL = *F*^−1^(*p*; μ, σ^2^). The simulation involves 5,000 independent data sets for each set of parameters. We compared five approaches: *a*) direct estimation (Tobit regression) of MLEs (μ̂ and ς̂^2^) using Equation 1; *b*) multiple imputation with allowance for uncertainty in model parameters; *c*) single imputation based on a random fill-in value for each datum below the DL, using MLEs (μ̂ and ς̂^2^) from Equation 1; *d*) insertion of DL/2 for all data below the DL; and *e*) insertion of *E*[*Z*|*Z* < DL] for data below the DL with the expected value based on the MLEs (μ̂ and ς̂^2^) from Equation 1. For approaches *b*) through *e*), estimators are the mean and variance of the logarithm of the observed and imputed data, with adjustment for multiple imputation in *b*). We compare results with estimates based on complete data.

For the NHL example, we use SAS (SAS System for Windows, version 8.2; SAS Institute Inc., Cary, NC) to generate bootstrap samples, fit linear regressions (PROC REG), solve log-likelihood Equations 1 and 2 (PROC LIFEREG), and combine results from multiple data sets (PROC MIANALYZE). The simulation was conducted using MATLAB (version 7.0; MathWorks Inc., Natick, MA).

## Results

We limited results to four insecticides, which exhibited various types and proportions of missing data: propoxur and carbaryl, both carbamate insecticides; chlorpyrifos, an organophosphate; and α-chlordane, an organochlorine.

### Regression analysis in control subjects.

**Figure 1:**
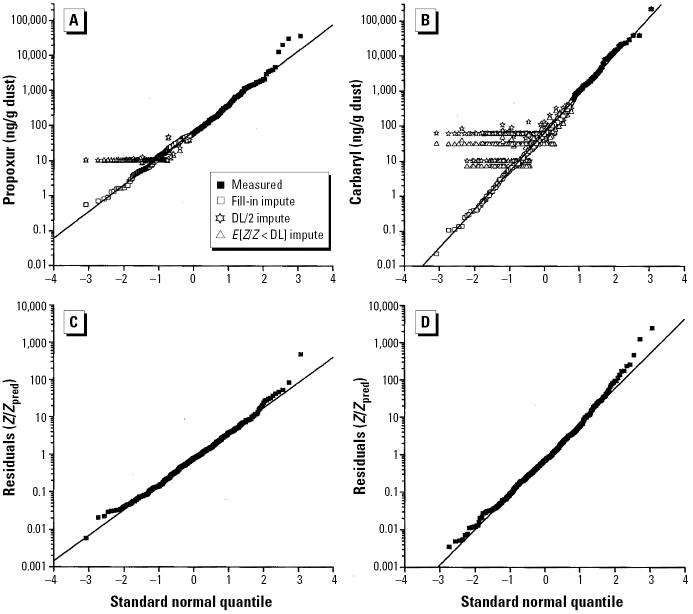
Plots under a log-normal distribution of quantiles of environmental measurements of (*A*) propoxur and (*B*) carbaryl, and of regression residuals of measurements (*Z*) and predicted values (*Z*_Pred_) after accounting for covariates for (*C*) propoxur and (*D*) carbaryl. The AMs, GMs, and GSDs are computed from imputed data. (*A*) AM = 456.6; GM = 65.6; GSD = 6.0. (*B*) AM = 1503.0; GM = 64.0; GSD = 14.0. (*C*) AM = 3.5; GM = 0.9; GSD = 2.0. (*D*) AM = 15.1; GM = 0.9; GSD = 2.6.

After omitting subjects’ missing questionnaire data, there are 478 control subjects with carpet dust measurements and all regression variables. The percentages of measurements below DLs or known only within bounds vary from 25.7% for propoxur to 67.0% for carbaryl (Table 1). The arithmetic mean (AM), geometric mean (GM), and geometric standard deviation (GSD), with fill-in imputations for interval-measured values, indicate that concentrations for the individual analytes varied substantially. For carbaryl and α-chlordane, the GM falls within the range of missing data. Figure 1A and B show quantile plots for measurements of propoxur and carbaryl and reveals good concordance with a log-normal distribution; Figure 1A and B show values used for imputation based on DL/2, denoted by stars, and the conditional expected value, denoted by triangles. For carbaryl, DL/2 values are nearly twice the conditional expected values. By construction, the fill-in values conform to the estimated distribution.

In several instances, estimates for the various types of pests treated differ substantially, particularly for analytes with a high percentage of missing data. The multiplicative standard errors for the replacement approaches (i.e., inserting DL/2, *E*[*Z*|LB < *Z* < UB], or a fill-in value) are smaller than standard errors from the multiple imputation approach and direct estimation. The smaller standard errors result from an inadequate account of missing data and result in CIs that are too narrow and inflated type I error rates. Table 2 shows several variables that do not achieve traditional significance levels when imputation is taken into account. In some instances, there are marked differences in estimates. Estimated increases in carpet dust levels of α-chlordane among subjects treating for termites are 2.6- and 3.1-fold based on DL/2 insertion and fill-in methods, respectively, and 3.7-fold based on multiple imputation and direct estimation approaches.

Comparing the two fill-in approaches, standard errors are smaller when the covariate information is included than when covariate information is omitted. Fill-in values are obtained from regression models by sampling from LN(β̂*^t^X_i_*, ς̂^2^). Figure 1C and D show quantile plots of residuals, that is, from exp[log(*Z*) – β̂*^t^X*]for each subject. Although GMs of the residuals are close to the expected value of 1.0 for the error distributions, plots suggest a slight underprediction at extreme values for propoxur and carbaryl.

### Simulation study.

For the simulation study, we set μ = 0 and σ^2^ = 1 without loss of generality and present results for *n* = 50, 100, 200, and 400 and with DLs such that the expected proportions of values below the DL are *p* = 10, 30, 50, and 70%. With 5,000 repetitions, the standard error for coverage of 95% CIs is 0.003. Table 3 shows that estimates of μ based on Tobit regression, multiple imputation, and single impute fill-in approaches are generally unbiased. Insertion of DL/2 or *E*[*Z*|*Z* < DL] results in substantial bias unless the proportion of missing data is small, ≤ 10%. Table 3 also shows coverage of the 95% CI for the estimate of μ. In comparison with complete data, Tobit regression and the multiple imputation approaches are the only methods that achieve nominal coverage over a broad range of simulation parameters, although the multiple imputation begins to degrade when more than about 50% of the measurements are below DLs. The single imputation approach results in anomalous CIs when about ≥ 30% of the data are below DLs.

## Discussion

Results of our analysis of use of pesticide products in and around the home and pesticide residues in carpet dust and of the simulation study suggest that the method of imputation of missing environmental measurement data can substantially affect estimation of effects and statistical inference. The practice of inserting a single value, such as DL/2 or the conditional expected value *E*[*Z*|*Z* < DL] or by analogy DL/√2 , is ill-advised unless there are relatively few measurements below DLs. The use of a single imputation to fill in missing data is unbiased or minimally biased quite generally but suffers from inaccurate estimates of variance and, consequently, CIs that are too narrow, particularly when missing data exceed about 30%. The best protection against biased inference in the presence of nonignorable missing data is the use of multiple imputation, although with a high proportion of values below the DL, a large number of measurements are needed. It is worth reiterating, however, that multiple imputation is necessary only if explicit values are needed for measurements below DLs. If the purpose is to estimate regression parameters, then procedures for truncated data, such as Tobit regression, are nominal (Little and Rubin 1987).

In environmental monitoring, estimation of distributional parameters is often problematic because of limited numbers of measurements and an inability to evaluate distributional forms precisely. With 5–15 measurements, MLEs can be biased (Gleit 1985), suggesting the need for more robust approaches (Helsel 1990). With epidemiologic data, which usually include hundreds or thousands of measurements, MLEs are unbiased and fully efficient (Gilliom and Helsel 1986), and more detailed regression analyses are feasible.

When analyzing environmental data on pesticides, Moschandreas et al. used a fill-in imputation approach that applied the “best-fitting” probability distribution for values above a DL (Helsel 1990; Moschandreas et al. 2001a, 2001b), although Helsel and Hirsch (1991) had cautioned that the approach should be used primarily for estimating summary statistics. The approach we outline permits multiple DLs, incorporates regression parameters, and applies multiple imputation to account correctly for interval-measured data and to allow unbiased inference. However, our simulation study suggests that the fill-in approach may be quite adequate when measurements below the DL account for less than about 30% of the data.

The Tobit regression and multiple imputation approaches assume that the limits of detection are fixed and known in advance. In our example, we are justified in assuming DLs are fixed for type I missing measurements, but not for type II and III missing data where DLs depend on the amount of interfering compounds and are random variables. If the DL is not known, an estimate of its value is the minimum order statistic of the observed measurements—that is, the smallest measured value. Simulations suggest that for a random DL, estimates remain unbiased but variances are underestimated (Zuehlke 2003). Thus, CIs in Table 2 may be too narrow. However, relative to other sources of uncertainty that arise in the collection and handling of carpet dust samples, and the accuracy of questionnaire information, additional uncertainty induced by random DLs for type II and III missing values is likely small.

Environmental data are frequently well approximated by a log-normal distribution, and our data on concentrations of pesticide analyte in carpet dust are consistent with this assumption. Equations 1 and 2 remain valid for more general distributions, although estimation of parameters may be more problematic and necessitate potentially computer-intensive search algorithms. Validity of parameter estimates and their variances depend, of course, on the correct choice of error distribution. Our simulation study was based on a correct distributional form; however, misspecification of the probability model can lead to markedly biased results (Paarsch 1984). In the absence of knowledge about the error distribution, semiparametric and nonparametric methods have been proposed (Austin 2002a; Chay and Powell 2001; DiNardo and Tobias 2001). Bayesian approaches have also been suggested in the Tobit regression context (Austin 2002b). A reviewer suggested considering the set of measurements of a subject as a vector of multivariate outcomes, so that the covariance structure among the analytes could provide information for the imputation process. In our example, this requires the assumption that the logarithms of the measurements are multivariate normally distributed. The suggestion, however, adds complexity as the number of analytes increases, and additional work is needed to evaluate its practical feasibility.

The motivation for this work arose from the analysis of pesticide analytes in carpet dust and use of pesticide products in and around the home. However, data with DLs arise in a variety of settings, including upper DLs from health-care–related questionnaire data (Austin 2002a) and psychological profile scores, such as the Fagerstrom test for nicotine dependence (Fagerstrom and Schneider 1989; Heatherton et al. 1991) and lower DLs in radiation film badge measurements (Gilbert et al. 1996; Kerr 1994).

In summary, with epidemiologic data, our analyses indicate that unless there are very few measurements below DLs (< 5–10%), inserting DL/2, *E*[*Z*|*Z* < DL], or any single value to impute missing measurement data is not advisable. Further, inserting a randomly selected fill-in value is also inadvisable, unless the proportion of missing data is less than about 30%. Multiple imputation of missing data is the best approach of ensuring unbiased estimates of effects and nominal CIs.

## Figures and Tables

**Table 1:** Percentage of measurements below DLs or known only within bounds and AMs, GMs, and GSDs based on fill-in values from a single imputation (data on 478 control subjects).

	Measurements known only within bounds								
	Type I		Type II		Type III		Dust (ng/g)		
Insecticide	Percent	Range	Percent	Range	Percent	Range	AM	GM	GSD
Propoxur	21.1	(0–46.0)	2.9	(0–65.0)	1.7	(21.1–75.7)	456.6	65.6	6.0
Carbaryl	37.9	(0–260.0)	11.1	(0–268)	18.0	(20.7–694.8)	1503.0	64.0	14.0
Chlorpyrifos	28.2	(0–77.4)	0.2	(0–20.9)	0.0	—	893.1	105.6	8.3
α-Chlordane	60.9	(0–44.7)	0.0	—	0.4	(20.8–29.1)	90.7	11.6	8.0

Types of missing measurements are as follows: no analyte detected and no interfering compound (I), no analyte detected but with an interfering compound present (II), and analyte and interfering compounds both present (III). The range for the DLs reflects the minimum of LBs and the maximum of UBs for the nondetected measurements.

**Table 2:** Proportional increase in analyte concentration in carpet dust (ng/g) for selected uses.

		Crawling insects		Flying insects		Fleas/ticks		Termites		Lawn/garden insects	
Insecticide, imputation approach*^a^* method	Adjustment	exp(β )	SE	exp(β )	SE	exp(β )	SE	exp(β )	SE	exp(β )	SE
Propoxur											
DL/2	No	1.426*^b^*	1.167	0.987	1.144	1.231	1.153	1.145	1.219	0.756*^b^*	1.151
*E*[*Z*|LB < *Z* < UB]	No	1.432*^b^*	1.170	0.986	1.147	1.231	1.156	1.135	1.223	0.751*^b^*	1.154
Fill-in	No	1.459*^b^*	1.189	0.966	1.163	1.225	1.173	1.072	1.249	0.737*^c^*	1.171
Fill-in	Yes	1.511*^b^*	1.182	1.030	1.157	1.251	1.166	1.209	1.239	0.687*^b^*	1.165
Multiple impute	Yes	1.487*^b^*	1.196	1.016	1.165	1.247	1.170	1.082	1.244	0.704*^b^*	1.173
Direct estimate	Yes	1.503*^c^*	1.276	0.994	1.235	1.245	1.250	1.090	1.363	0.714	1.249
Carbaryl											
DL/2	No	0.853	1.201	0.661*^b^*	1.173	1.560*^b^*	1.185	1.129	1.266	1.660*^b^*	1.183
*E*[*Z*|LB < *Z* < UB]	No	0.849	1.226	0.629*^b^*	1.194	1.703*^b^*	1.208	1.199	1.300	1.746*^b^*	1.205
Fill-in	No	0.830	1.311	0.591*^b^*	1.265	1.812*^b^*	1.285	1.486	1.417	1.735*^b^*	1.282
Fill-in	Yes	0.940	1.274	0.432*^b^*	1.235	2.337*^b^*	1.252	1.538	1.366	1.779*^b^*	1.249
Multiple impute	Yes	0.826	1.338	0.508*^b^*	1.272	2.047*^b^*	1.313	1.326	1.490	1.950*^b^*	1.351
Direct estimate	Yes	0.785	1.499	0.512*^c^*	1.413	2.180*^b^*	1.452	1.281	1.651	2.115*^b^*	1.444
Chlorpyrifos											
DL/2	No	1.578*^b^*	1.209	0.779	1.181	1.264	1.182	1.581*^c^*	1.276	0.759	1.188
*E*[*Z*|LB < *Z* < UB]	No	1.620*^b^*	1.218	0.771	1.188	1.300	1.190	1.613*^c^*	1.288	0.746	1.196
Fill-in	No	1.917*^b^*	1.243	0.757	1.210	1.389*^c^*	1.212	1.669*^c^*	1.322	0.713*^c^*	1.219
Fill-in	Yes	1.745*^b^*	1.244	0.740	1.210	1.383*^c^*	1.212	1.631*^c^*	1.323	0.731	1.219
Multiple impute	Yes	1.770*^b^*	1.252	0.763	1.223	1.401*^c^*	1.223	1.689*^c^*	1.336	0.708	1.234
Direct estimate	Yes	1.796*^c^*	1.378	0.740	1.323	1.392	1.327	1.698	1.492	0.702	1.338
α-Chlordane											
DL/2	No	0.966	1.129	0.938	1.112	0.910	1.118	2.626*^b^*	1.168	1.091	1.117
*E*[*Z*|LB < *Z* < UB]	No	0.954	1.153	0.925	1.132	0.894	1.140	3.031*^b^*	1.199	1.110	1.138
Fill-in	No	1.060	1.230	0.828	1.198	0.868	1.210	3.110*^b^*	1.303	1.079	1.208
Fill-in	Yes	0.762	1.206	0.927	1.177	0.908	1.188	3.898*^b^*	1.271	1.293	1.186
Multiple impute	Yes	0.852	1.363	0.915	1.235	0.804	1.202	3.686*^b^*	1.290	1.169	1.270
Direct estimate	Yes	0.858	1.379	0.919	1.316	0.803	1.339	3.666*^b^*	1.442	1.211	1.334

Entries are exponentials of parameter estimates (β) and their SEs from linear regression models of the logarithm of pesticide analyte on age, sex, race, education, study site, season, and pesticide use variables. Regression models also included year house was built (propoxur, carbaryl, α-chlordane), type of home (carbaryl), and presence of oriental rugs (α-chlordane).

a See “Materials and Methods” for a description of methods; adjusted imputation includes regression variables.

b 95% CI excludes 1.

c 90% CI excludes 1.

**Table 3:** Results of simulation study of imputation approaches*^a^* for log-normally distributed data with μ = 0 and σ^2^ = 1 with a DL (entries are means of 5,000 repetitions).

Sample size (no.)	Percent < DL	Complete data	Tobit analysis	Multi-impute using (μ̂, σ̂^2^)	Single impute using (μ̃, σ̃^2^)	Insert DL/2	Insert *E*[*Z*|*Z* < DL]
50							
Estimate of μ	10.0	0.002	0.000	−0.003	−0.003	−0.020	0.007
	30.0	0.002	−0.003	−0.003	−0.004	−0.017	0.032
	50.0	0.002	−0.004	−0.003	−0.003	0.052	0.073
	70.0	0.002	−0.006	−0.005	−0.002	0.229	0.143
Coverage of 95% CI	10.0	0.947	0.944	0.943	0.943	0.943	0.942
	30.0	0.947	0.949	0.938	0.928	0.942	0.928
	50.0	0.947	0.953	0.928	0.876	0.938	0.832
	70.0	0.947	0.931	0.895	0.707	0.280	0.520
100							
Estimate of μ	10.0	0.003	0.002	0.000	0.000	−0.019	0.009
	30.0	0.003	0.001	0.000	0.000	−0.015	0.034
	50.0	0.003	0.000	0.000	−0.001	0.055	0.076
	70.0	0.003	−0.006	−0.004	−0.002	0.232	0.142
Coverage of 95% CI	10.0	0.944	0.945	0.940	0.940	0.943	0.942
	30.0	0.944	0.949	0.938	0.929	0.942	0.914
	50.0	0.944	0.948	0.922	0.870	0.910	0.781
	70.0	0.944	0.940	0.904	0.721	0.036	0.440
200							
Estimate of μ	10.0	−0.001	−0.002	−0.002	−0.002	−0.023	0.006
	30.0	−0.001	−0.003	−0.003	−0.003	−0.019	0.031
	50.0	−0.001	−0.002	−0.002	−0.002	0.052	0.074
	70.0	−0.001	−0.003	−0.001	−0.002	0.229	0.142
Coverage of 95% CI	10.0	0.952	0.950	0.951	0.950	0.941	0.946
	30.0	0.952	0.955	0.936	0.926	0.940	0.904
	50.0	0.952	0.948	0.925	0.874	0.877	0.708
	70.0	0.952	0.947	0.914	0.725	0.000	0.306
400							
Estimate of μ	10.0	0.001	0.001	0.001	0.001	−0.021	0.008
	30.0	0.001	0.000	0.000	0.000	−0.017	0.034
	50.0	0.001	0.001	0.001	0.001	0.053	0.076
	70.0	0.001	0.000	0.000	0.000	0.230	0.144
Coverage of 95% CI	10.0	0.954	0.954	0.952	0.951	0.931	0.949
	30.0	0.954	0.948	0.938	0.928	0.941	0.874
	50.0	0.954	0.954	0.927	0.880	0.776	0.545
	70.0	0.954	0.947	0.914	0.723	0.000	0.128

a Parameter estimation using observed data with DLs (Tobit analysis), (μ̂, σ̂^2^) multiple imputation with allowance for uncertainty in model parameters using (μ̃, σ̃^2^), a single imputation using (μ̂, σ̂^2^), the insertion of DL/2, and insertion of the expected value conditional on being below the DL, *E*[*Z*|*Z* < DL].
